# Supplementary figures and images for: Cortical feedback and gating in odor discrimination and generalization
Source: PLoS Comput Biol. 2021 Oct 11;17(10):e1009479. doi: 10.1371/journal.pcbi.1009479 (PMC8530364; doi:10.1371/journal.pcbi.1009479)

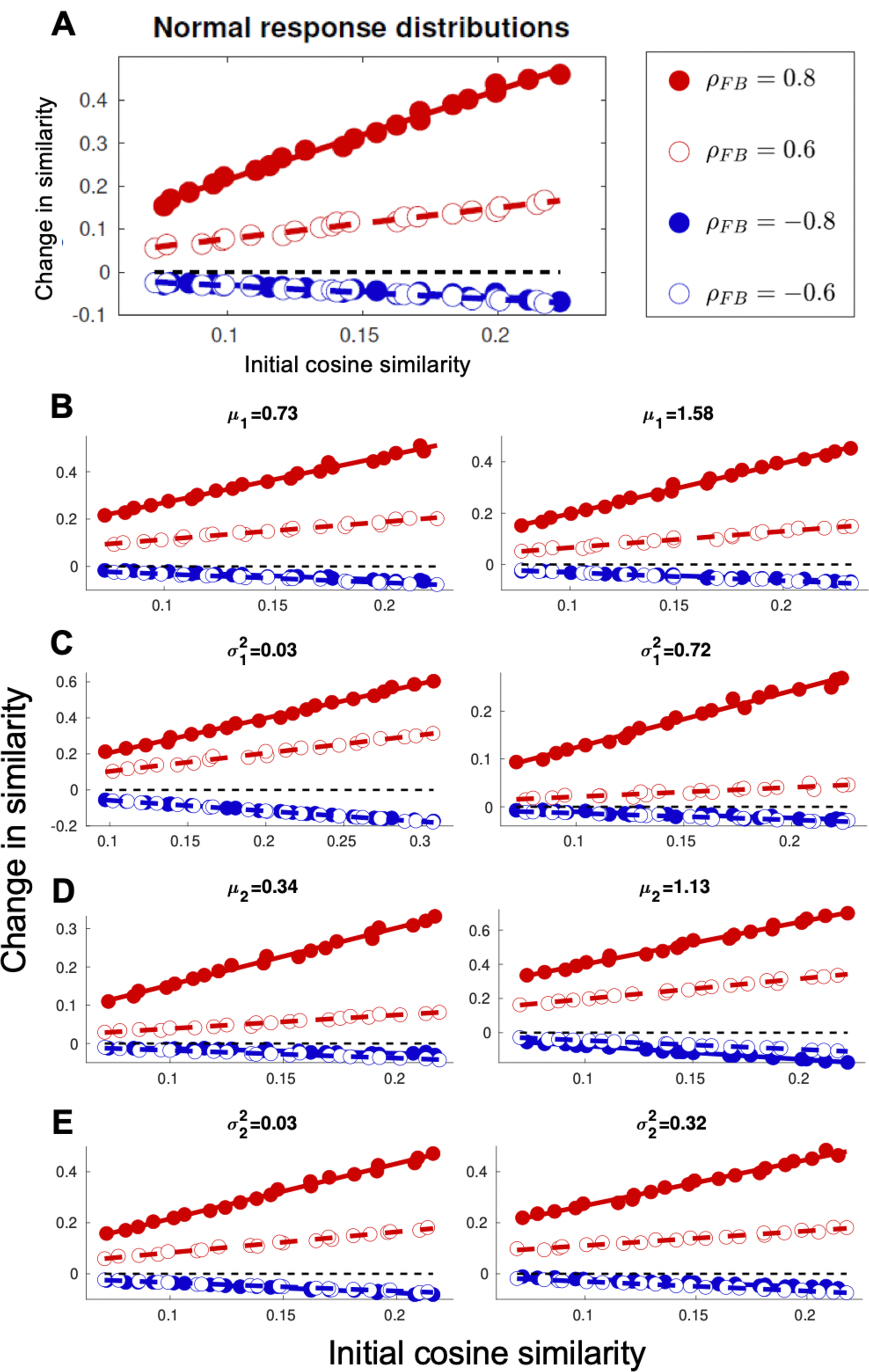

Supplement: S1 Fig — (A) Results obtained from the statistical model with mean and variance of the normal distributions: μ1 = 1.15, σ12=0.42 for odor inputs, and μ2 = 0.57, σ22=0.28 for feedback inputs. The cortical threshold is set to θc = 1.6 to give cortical activation of 10% [25]. Datapoint obtained by averaging the results for 10 randomly generated pairs of odor inputs with close similarity values. Different markers indicate different feedback conditions. Filled red/blue circles: the two feedback scenarios of Fig 2, corresponding to ρFB = 0.8 and ρFB = −0.8, respectively. Note that |ρFB ≠ 1| due to variability in the amplitude of the feedback strength ΔR. Empty red/blue circles: two intermediate conditions, corresponding to 50% of modules receiving feedback, of which 75% are shared between the two odors. Of the feedback-targeted modules, 75% receive inhibitory feedback for both odors in the first case (red, ρFB = 0.6), and 75% / 12.5% receive inhibitory feedback for the first/second odor in the second case (blue, ρFB = −0.6) (B–E) The parameters of the distributions are varied one by one with respect to (A). Results are robust against changes in (B, C) the mean and variance, respectively, of the distributions of module responses to odor inputs, and (D, E) the mean and variance, respectively, of the distributions of module responses to feedback inputs. As in (A), each datapoint is obtained by averaging the results for 10 randomly generated pairs of odor inputs with close similarity values. Different markers indicate different feedback conditions. Filled red/blue circles: the two feedback scenarios of Fig 2, corresponding to ρFB = 0.8 and ρFB = −0.8 in (B–C), ρFB = 0.75 and ρFB = −0.75 in (D, left), ρFB = 0.94 and ρFB = −0.94 in (D, right), ρFB = 0.92 and ρFB = −0.92 in (E, left), ρFB = 0.72 and ρFB = −0.72 in (E, right). Empty red/blue circles: two intermediate conditions, corresponding to 50% of modules receiving feedback, of which 75% are shared between the two odors. Of the feedb [file pcbi.1009479.s001.tif]

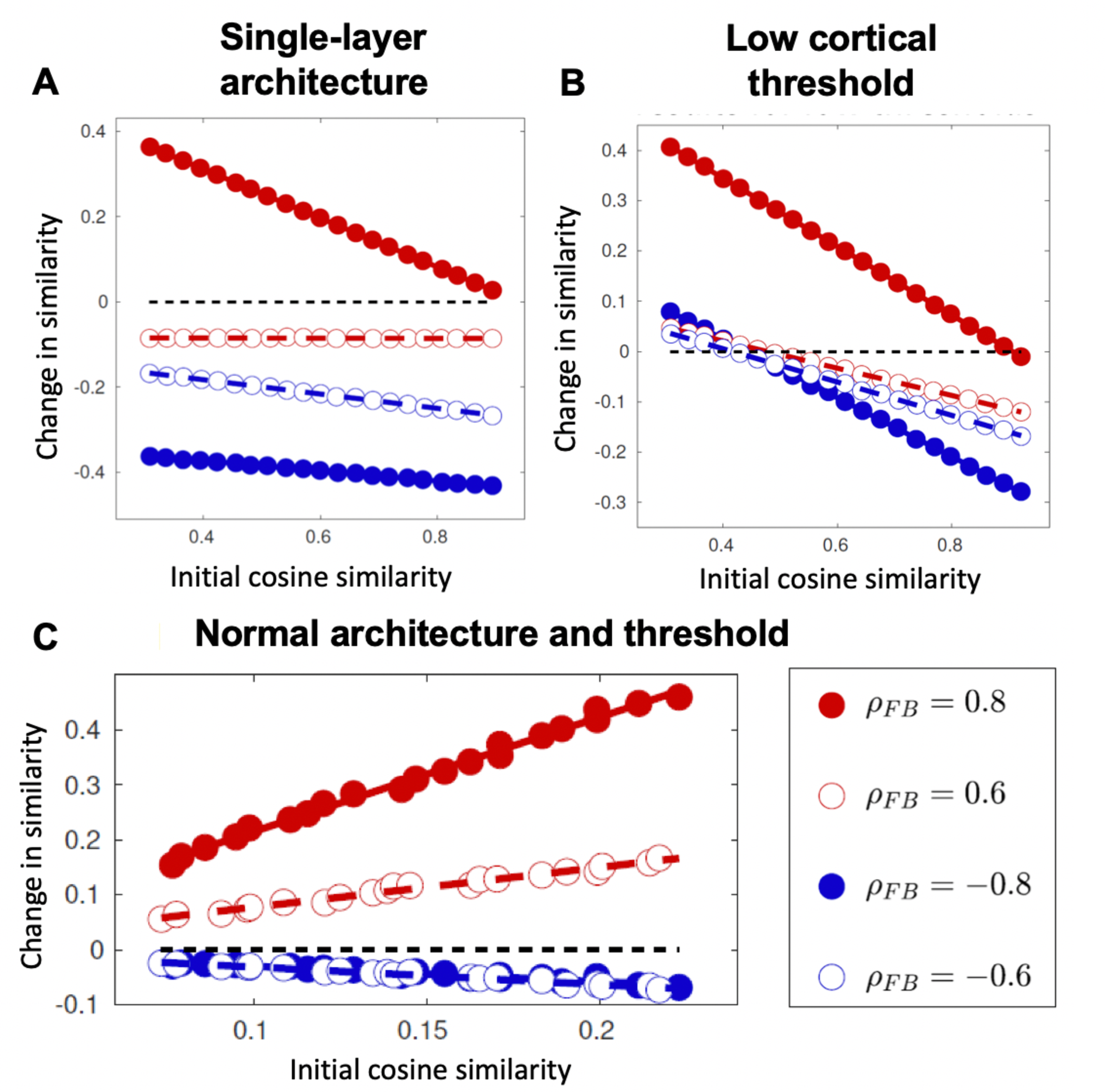

Supplement: S2 Fig — The statistical effects induced by feedback in a single-layer architecture or with low cortical threshold are qualitatively different from those arising in a two-layer model with a high-threshold transfer function. (A) With only one layer, pattern convergence can be achieved only when the feedback similarity is very high and decreases with increasing odor similarity (red). Moderately correlated and anticorrelated feedback induce similar effects (empty red and blue circles). Same feedback conditions as in (C): Filled red/blue circles for the two extreme feedback scenarios with ρFB = 0.8 and ρFB = −0.8, respectively; empty red/blue circles for the two intermediate feedback conditions, with ρFB = 0.6 and ρFB = −0.6, respectively. (B) Same conditions as in (C) except with lower cortical threshold. The trend reversal is similar to that seen in the analytical framework (Fig 5A). (C) S1A Fig demonstrating results from a normal two-layer, high-threshold architecture for comparison. (TIF) [file pcbi.1009479.s002.tif]

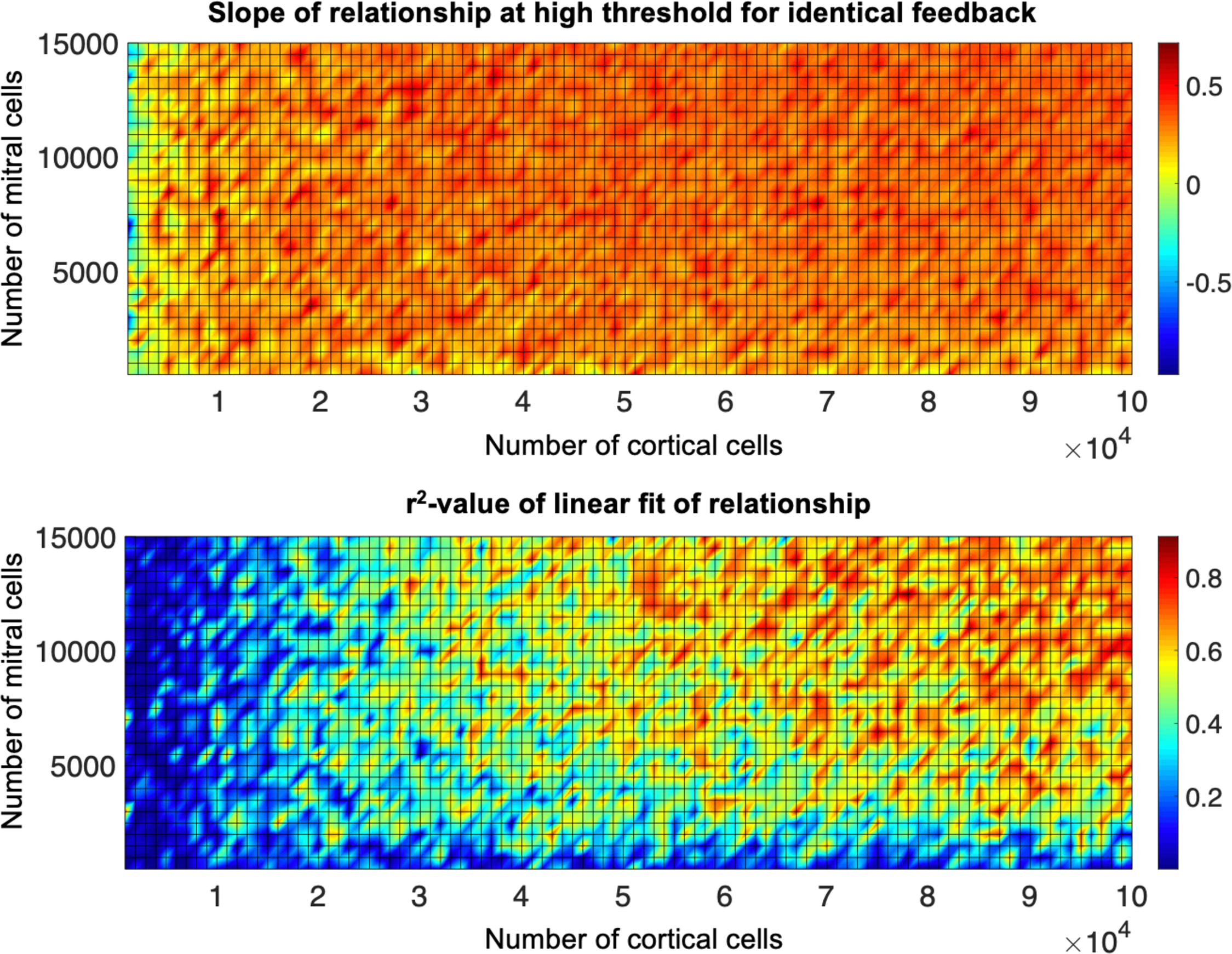

Supplement: S3 Fig — A robustly positive slope of the relationship between initial similarity and change in similarity at high threshold is only achieved for a sufficient number of MCs and bulb modules (i.e., cortical cells). For different numbers of M mitral cells and K cortical cells, we simulated presentation of the same positive feedback for different pairs of odors and then measured the slope of the relationship between initial similarity and change in similarity at high threshold. Although positive slope was achieved for relatively low numbers of MCs and cortical cells, this relationship did not achieve a consistently high r2 value without approximate values of M > 8000 and K > 80000. For all simulations, fodor = 0.12, pFB = 0.08 (fraction of feedback-targeted MCs), and q = 0.07. (TIF) [file pcbi.1009479.s003.tif]

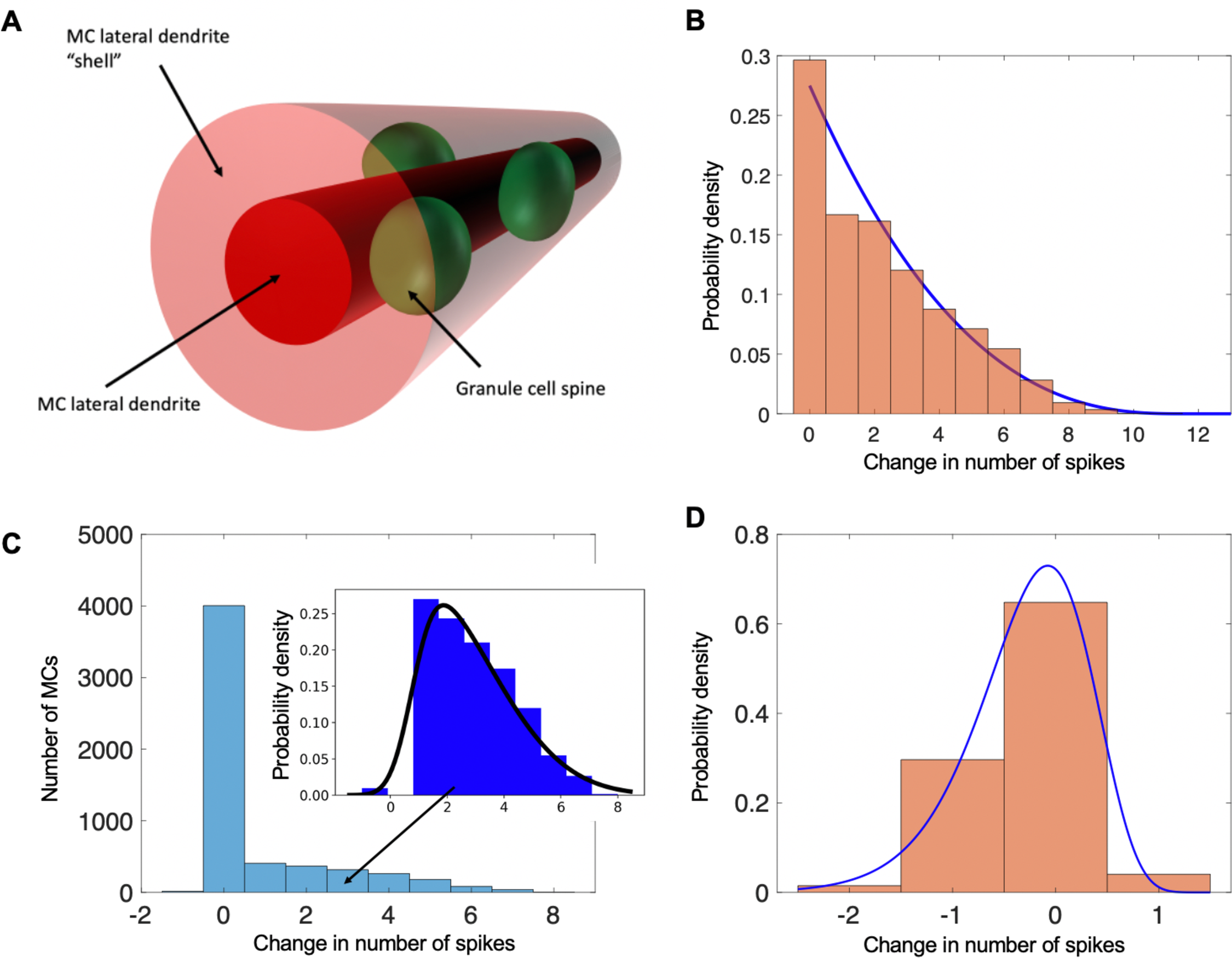

Supplement: S4 Fig — (A) Depiction of the relevant volumes for calculating MC-GC connectivity (B) Distribution of MC firing rates due to odor input (C) Distribution of changes in MC firing rates due to excitatory feedback. Inset shows the skew normal distribution that was sampled if the change was not equal to 0 (see “Firing rate distributions”) (D) Distribution of changes in odor-receiving MC firing rates due to excitatory feedback to GCs. Note that the lognormal distribution has been shifted to match the range of the data. The parameters of the simulations for the distributions in B–D are provided in the text of the Methods (“Neuronal and network dynamics; Firing rate distributions”). (TIF) [file pcbi.1009479.s004.tif]

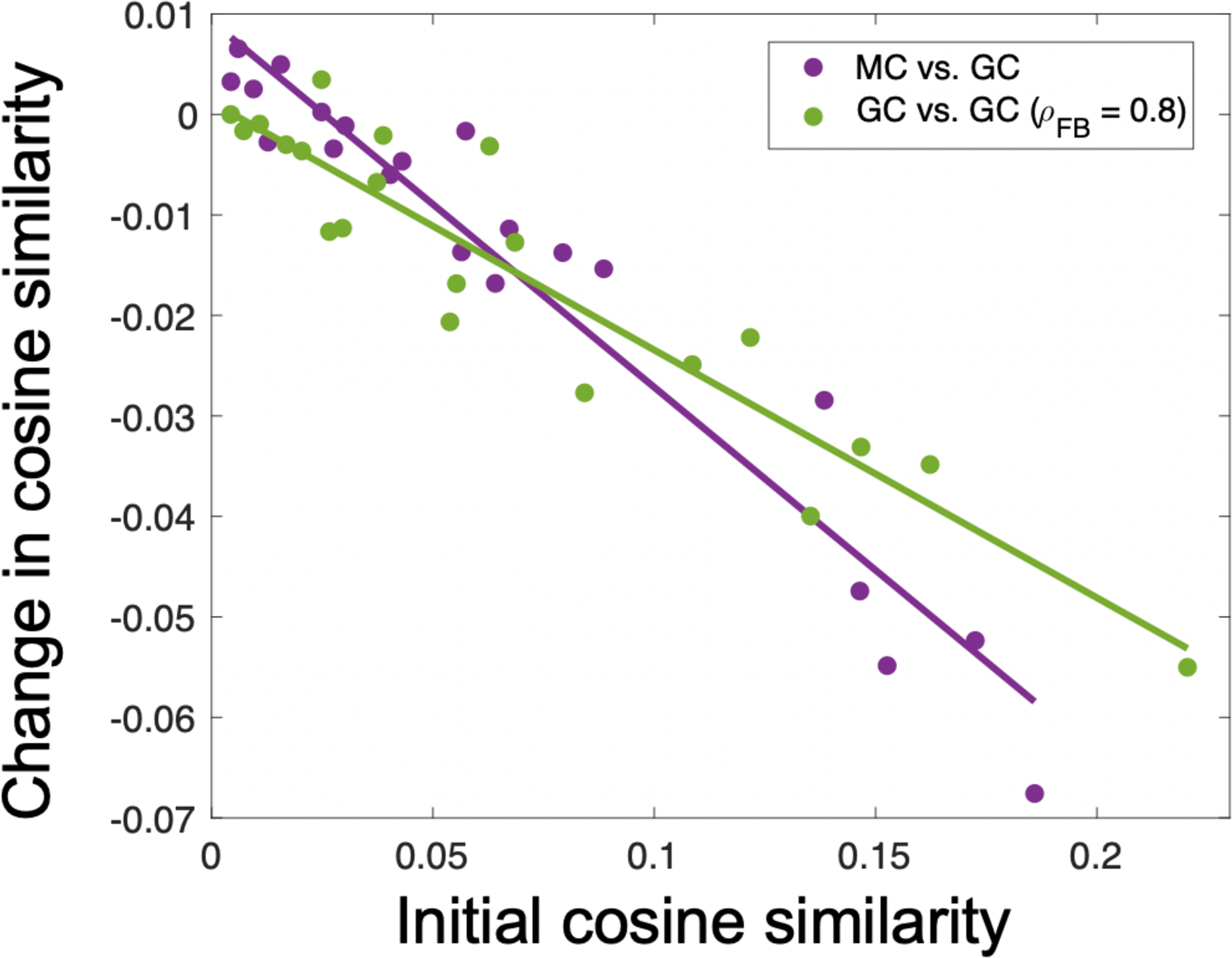

Supplement: S5 Fig — For high cortical thresholds, pattern divergence occurred when excitatory feedback was presented for one odor and inhibitory for the other (purple line), or when highly correlated inhibitory feedback was presented for both odors (green line). In all cases, we simulated 10,000 MCs grouped into 500 glomeruli and 100,000 cortical cells, each sampling 7% of the MCs, with odor targeting 12% of the glomeruli, positive feedback targeting 8% of the MCs, and negative feedback targeting all MCs. (TIF) [file pcbi.1009479.s005.tif]

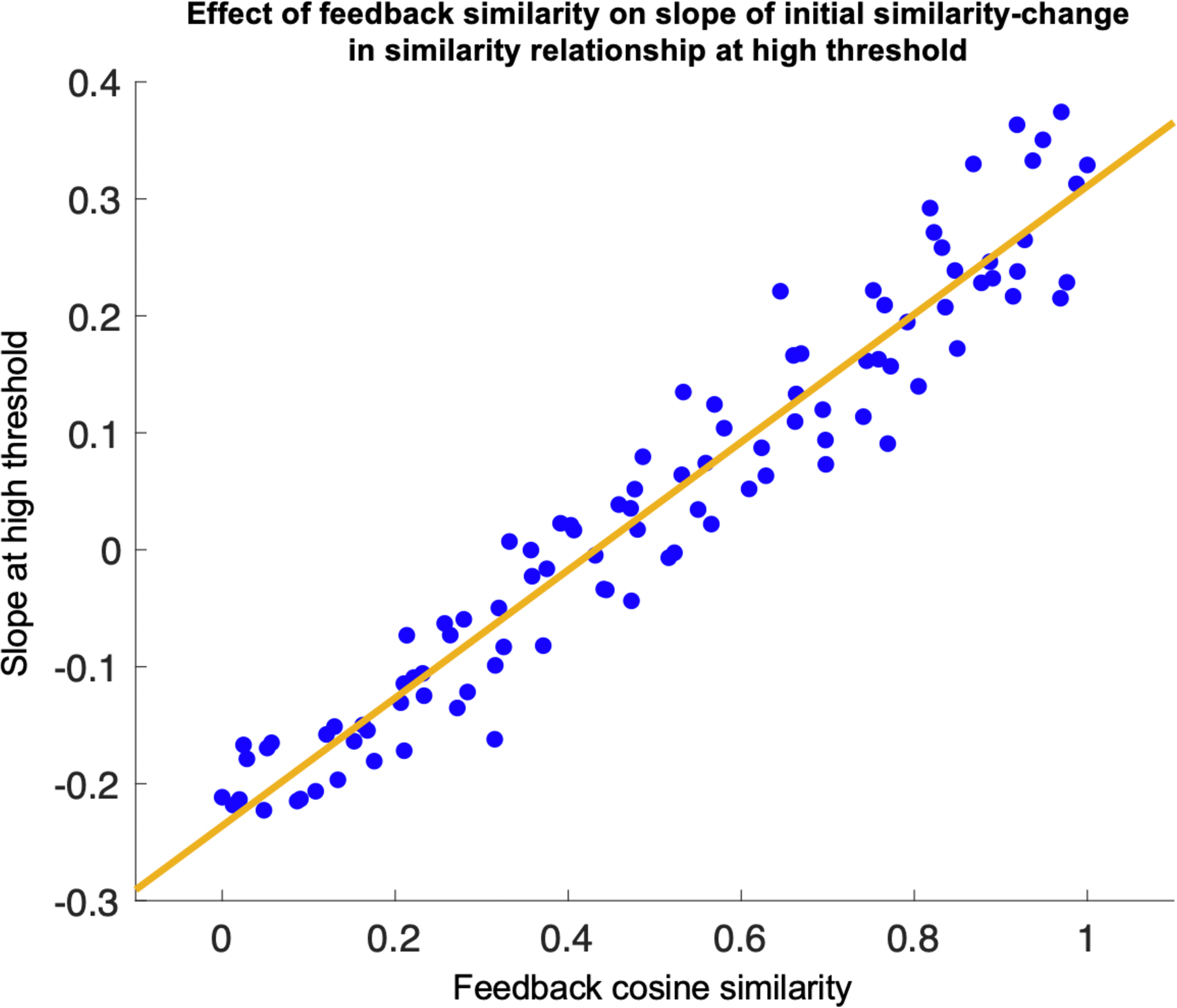

Supplement: S6 Fig — Sufficiently correlated positive feedback produces a proportional relationship between initial similarity and change in similarity. We generated a range of feedback similarities for pairs of excitatory feedback vectors. We found that the slope of the relationship between initial similarity and change in similarity at high threshold varied linearly with the feedback similarity (slope = 0.5469, r2 = 0.9322). Thus, for feedback vectors with significant similarity, the relationship between initial similarity of two odor representations and the change in similarity of those representations following feedback is positive. For all simulations, M = 10000, K = 100000, fodor = 0.12, pFB = 0.08, G = 500, and q = 0.07. (TIF) [file pcbi.1009479.s006.tif]
