# Supplementary material for: Cortical feedback and gating in odor discrimination and generalization
Source: PLoS Comput Biol. 2021 Oct 11;17(10):e1009479. doi: 10.1371/journal.pcbi.1009479 (PMC8530364; doi:10.1371/journal.pcbi.1009479)
Supplement: S1 Text — (PDF) [file pcbi.1009479.s007.pdf]

S1 Text Pattern convergence and divergence for normal distributions of module responses: numerical results. We noted that the firing rate of modules should be determined by the sum of the firing rates of the component mitral cells. Thus, by the central limit theorem, the module firing rates should be normally distributed. Likewise, feedback affects individual mitral cells, and the resulting change in the firing rate of a module is determined by the sum of these changes for the component mitral cells. Thus, the effect of feedback on the module firing rates should also be normally distributed. We then simulated $N = 10,000$ cortical units receiving inputs from the corresponding number of bulb modules, and responding to two odors A and B. Before feedback, we took the responses of bulb modules to be normally distributed for each odor. We controlled initial odor similarity by changing the fraction of modules that responded to both odors as opposed to only one. We then modeled the effects of contextual feedback by adding normally distributed firing rate changes to the modules. The mean and standard deviation of the distributions were picked so that the range of module responses would be comparable to the previous analytical computations. Additionally, the hard threshold for cortical activation in the analytical model was replaced with a sigmoid acting on the olfactory bulb module responses with a soft threshold at θ_c. These simulations of the statistical model produced trends in pattern convergence and divergence that were qualitatively similar to those derived analytically. We also were able to show that these results were robust to broad changes in parameters (S1 Fig).
