# Supplementary material for: Cortical feedback and gating in odor discrimination and generalization
Source: PLoS Comput Biol. 2021 Oct 11;17(10):e1009479. doi: 10.1371/journal.pcbi.1009479 (PMC8530364; doi:10.1371/journal.pcbi.1009479)
Supplement: S2 Text — (PDF) [file pcbi.1009479.s008.pdf]

S2 Text The necessity of a nonlinear transfer function with high cortical threshold in simulations of the statistical model. Our results depend critically on a two-layer architecture where activity in the bulb is modulated by feedback and then passed through a nonlinearity with a high threshold in the cortex. To see this, we changed the nonlinearity to a linear function $f(R_i) = R_i$ (which effectively reduced the model to a single-layer) and studied how the similarity in bulb responses to odors changed due to feedback. As above, we assumed that these responses prior to feedback and the feedback-induced changes were both normally distributed, and we also maintained the same feedback similarities and statistics. We then computed the cosine similarity between bulb responses to odors A and B, before and after adding feedback. S2A Fig shows that the effects induced by feedback in the bulb are substantially different from those that arise in the cortical layer of the original model with high threshold (S2C Fig), whereas they are similar to those obtained in the original model with low thresholds (S2B Fig). In particular, in the single-layer model pattern convergence can be achieved only when the feedback similarity is very high, and even then decreases with increasing odor similarity, oppositely to the trend observed in the high-threshold original model. Thus, the form of pattern convergence of S2C Fig can only emerge in a network linked by a non-linear (high-threshold) transfer function.
