# Supplementary material for: Cortical feedback and gating in odor discrimination and generalization
Source: PLoS Comput Biol. 2021 Oct 11;17(10):e1009479. doi: 10.1371/journal.pcbi.1009479 (PMC8530364; doi:10.1371/journal.pcbi.1009479)
Supplement: Attachment — Submitted filename: Response_letter.pdf [file pcbi.1009479.s009.pdf]

Response to Referees

Reviewer 1

I really like the article, congratulations to the authors, it proposes a new vision of olfactory perception.

Thank you! We also felt that our model presents an exciting way to understand aspects of olfaction, and are glad that the referee agrees!

I would like to suggest in discussion to enlarge even only hypothetically:

i. **the interactions between the various odorants in the formation of the scent**

   Indeed, animals in the environment tend to encounter odor mixtures rather than isolated odors. In fact, our model only depends on the induced responses in the bulbar network rather than what combination of odors might be responsible for those responses. Thus, the model generalizes immediately to comparisons between single odorants or odorant mixtures, although for simplicity we described the model in terms of single odorants. Precisely the same analysis applies to odorant mixtures – we just have to excite the relevant glomeruli in relation to the concentrations of the odorants that activate the corresponding receptors. The activated glomeruli may overlap between mixtures or not, just like for single odorants. The only subtlety is that the activation of receptors by mixtures is a nonlinear function of the composition of the odor. There has been significant recent progress in understanding this nonlinearities from a biophysical perspective, e.g in a paper from our own group (Singh et al., PNAS 2019). Using these approaches would allow us to formulate the model in terms of the odorant concentrations in the mixtures, rather than directly in terms of the effective activations of glomeruli. This would be interesting to do in the future. We have added comments concerning these points in the Discussion.

ii. **the cross-modal interactions for example with the gustatory system**

   Indeed, interaction with other sensory modalities is a key feature of olfaction and sensation in general. We have added comments in the Discussion exploring how our model may assist in understanding such interactions.

iii. **and finally an important aspect since the ambition of the authors concerns also Alzheimer d. is to discuss how the model can interpret aging and especially olfactory phenotypes (see Oncotarget).**

   In addition to an expanded look at a potential way of investigating our model in the setting of Alzheimer's disease, we now also comment on the importance of aging in relation to olfaction and our model in the Discussion. Thank you for this suggestion.

minor points: I recommend a re-reading of the paper for minor adjustments, e.g. Ref # 84.

We have gone through the paper again; additionally, we have corrected reference 84. Thanks for pointing this out.

Reviewer 2

Within this work, the authors develop a model to predict convergence and divergence of cortical patterns in odor perception. This model contributes to understanding the effect of cortical feedback in odor perception. The authors made a great effort in explaining the concept behind their model and the anatomy it follows. The result is a very well-written and straightforward manuscript.

Thank you! We are glad that the paper is able to clearly convey our results.

As a general note, the authors should explain in more detail their hypothesis.

In short, our hypothesis was that unstructured feedback could induce changes in odor similarity that depended only on the relationship between feedback patterns rather than the exact constitution of individual patterns. We had initially expected that feedback would serve to make odor representations more uniform, i.e. for more similar odor patterns to more strongly separate during pattern divergence, and for less similar odor patterns to more strongly merge during pattern convergence. The introduction has been updated to clarify these initial expectations and our hypothesis.

I would also expect a more detailed explanation of the reason why they came up with the need to develop the model, what it can be useful for, and what it may bring to the field.

The use of a computational model allows for not only the investigation of large numbers of neurons, but also the direct control of the odor and feedback input distributions. As we see in the paper, both ultimately prove necessary to our results. Furthermore, our approach to modeling feedback provides a new perspective on its role in olfaction as well as on the interaction between central and peripheral brain regions in sensation in general. We have expanded on these points in the Introduction.

Specific points follow:

Line 77: You state that you have only minimal assumptions about statistics of odor and feedback input. Which are these assumptions? Why do you imply them? You explicit all the assumptions in the method section, but I would expect to explicit which kind of assumptions are you implying, without analyzing them in depth, as it has been done in the methods.

What we mean to say here is that our results hold as we relax assumptions concerning the distribution of odor and feedback inputs to the system. We initially made those assumptions to

derive analytical results, but then relaxed them in numerical simulations of the model (Fig.S1, Text S1). We have updated the paper to clarify.

Line 82: explain more in detail what do you mean by semantic context. This is the first time, and also the last one, you refer to a semantic context.

By semantic context, we simply mean the contextual information we alluded to previously in the introduction. The word "semantic" has been removed for clarity here, and we added an explanation in the first sentence of the paper of what we mean by contextual information.

Line 88: the authors mention Alzheimer's disease as a neurological condition that induces differences in cortical excitability. I would deepen the usefulness of your model from a clinical point of view.

In the Discussion we have added a suggestion for a clinical study, and our predictions for it.

Line 93: It is not clear why you refer to an "initial" model

By initial, we meant the statistical model being described, since we expand to the mechanistic model later in the paper. We have clarified the terminology.

Line 108: "context as the effect produced by cortical feedback" As you explicit in the abstract, I would also include in the introduction what do you mean by context.

Thank you for this suggestion. We now introduce a definition of context in the first line of the introduction for clarity.

Line 391: the authors state that in the presence of different odors and similar contexts, they would expect greater convergence. Please discuss more the initial hypothesis and the results,

We now clarify this in the Introduction.

Line 403: your work focused on the olfactory system. Why did you decide to focus on this particular perception and not on other systems?

Olfaction possesses a unique circuitry characterized by a reduced number of information-processing layers (i.e., the bulb and the piriform cortex) compared to other sensory systems, and a clearly defined relationship between these layers; this facilitates our investigation of feedback in a sensory system. Nonetheless, we hypothesize that other sensory modalities may also exhibit similar trends in pattern convergence and divergence as described for olfaction in our paper. We clarify this in the Discussion, section "Generality of the proposed mechanism across sensory modalities."

Line 487: how would you test your predictions in patients with AD?

We have introduced a design for a clinical study in the Discussion.

Line 522: “(i)” instead of “(ii)”

We have fixed this typo.
